# Gold Metallodrugs to Target Coronavirus Proteins: Inhibitory Effects on the Spike‐ACE2 Interaction and on PLpro Protease Activity by Auranofin and Gold Organometallics**

**DOI:** 10.1002/chem.202004112

**Published:** 2020-10-19

**Authors:** Maria Gil‐Moles, Uttara Basu, Rolf Büssing, Henrik Hoffmeister, Sebastian Türck, Agnieszka Varchmin, Ingo Ott

**Affiliations:** ^1^ Institute of Medicinal and Pharmaceutical Chemistry Technische Universität Braunschweig Beethovenstrasse 55 38106 Braunschweig Germany

**Keywords:** Auranofin, coronavirus, gold complexes, metallodrugs, SARS-CoV-2

## Abstract

Gold complexes have a long tradition in medicine and for many examples antirheumatic, anticancer or anti‐infective effects have been confirmed. Herein, we evaluated the lead compound Auranofin and five selected gold organometallics as inhibitors of two relevant drug targets of severe acute respiratory syndrome coronaviruses (SARS‐CoV). The gold metallodrugs were effective inhibitors of the interaction of the SARS‐CoV‐2 spike protein with the angiotensin converting enzyme 2 (ACE2) host receptor and might thus interfere with the viral entry process. The gold metallodrugs were also efficient inhibitors of the papain‐like protease (PLpro) of SARS‐CoV‐1 and SARS‐CoV‐2, which is a key enzyme in the viral replication. Regarding PLpro from SARS‐CoV‐2, the here reported inhibitors are among the very first experimentally confirmed examples with activity against this target enzyme. Importantly, the activity of the complexes against both PLpro enzymes correlated with the ability of the inhibitors to remove zinc ions from the labile zinc center of the enzyme. Taken together, the results of this pilot study suggest further evaluation of gold complexes as SARS‐CoV antiviral drugs.

## Graphical abstract

**Going for gold**! Gold complexes show inhibitory effects on selected SARS‐CoV‐2 protein targets. The strong inhibition of PLpro correlates with the ability of the complexes to remove zinc from the enzyme.

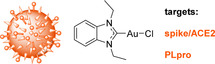

The current pandemic outbreak of the severe acute respiratory syndrome coronavirus 2 (SARS‐CoV‐2) has caused an unprecedented global health crisis with to date more than 29 million infected individuals.[^1^, ^2^] While the world struggles with the control of the fast outspread of this coronavirus and it's enormous impact on healthcare, economy and society, efforts to develop vaccines and therapeutics have been undertaken worldwide at a rate, which modern drug discovery has not witnessed ever. The lack of an effective antiviral drug for the treatment of the Coronavirus disease‐2019 (COVID‐19) has triggered major drug repurposing efforts; however, to this date no approved therapeutic has proven to have sufficient efficacy in the many ongoing clinical trials. The urgent development of new innovative drug candidates against SARS‐CoV‐2 is the most important mission that medicinal chemists are currently facing.

Regarding drug activity evaluation, several molecular pathways have been in the focus of the search for a possible COVID‐19 treatment based on strategies that had already been considered for the SARS‐CoV and Middle East respiratory syndrome MERS‐CoV outbreaks.^[3]^ Amongst others these include the entry of the coronavirus into the host cell (e.g. the interaction of TMPRSS2^[4]^ or ACE2 with spike proteins of the coronavirus^[5]^), the viral replication process in the host cell (e.g. the proteases 3CLpro^[6]^ and PLpro[^3^, ^7^, ^8^]), transcription, the nucleocapsid protein, or exocytosis of the new virion.[^3^, ^7^, ^9^]

Gold complexes have a long lasting history in medicine and have been used as disease modifying antirheumatic drugs (DMARDs) for the treatment of rheumatoid arthritis. Intensive research on other possible therapeutic applications of the lead compound Auranofin and other gold species has focused on anticancer and anti‐infective agents. The application of gold complexes as antiviral drugs has not been studied very intensively, although some promising results suggest a possible future use as human immunodeficiency virus (HIV) therapeutics.^[10]^

Here we report the results of a pilot study, in which we investigated the effects of Auranofin and selected experimental gold metallodrugs (see Figure 1) on two relevant coronavirus targets (spike protein, papain like protease, PLpro). Whereas **Au‐1**,^[11]^ **Au‐3**[^12^, ^13^] and **Au‐5** ^[14]^ were selected from our previous works on organometallic gold metallodrugs, **Au‐2** and **Au‐4** have not been reported before and their synthesis and characterization are described here. Complexes **Au‐1** to **Au‐5** are organometallics containing either a *N*‐heterocyclic carbene (NHC) or an alkynyl ligand. Complexes of these types have demonstrated promising activities in a fast increasing number of recent reports.^[15]^

**Figure 1:**
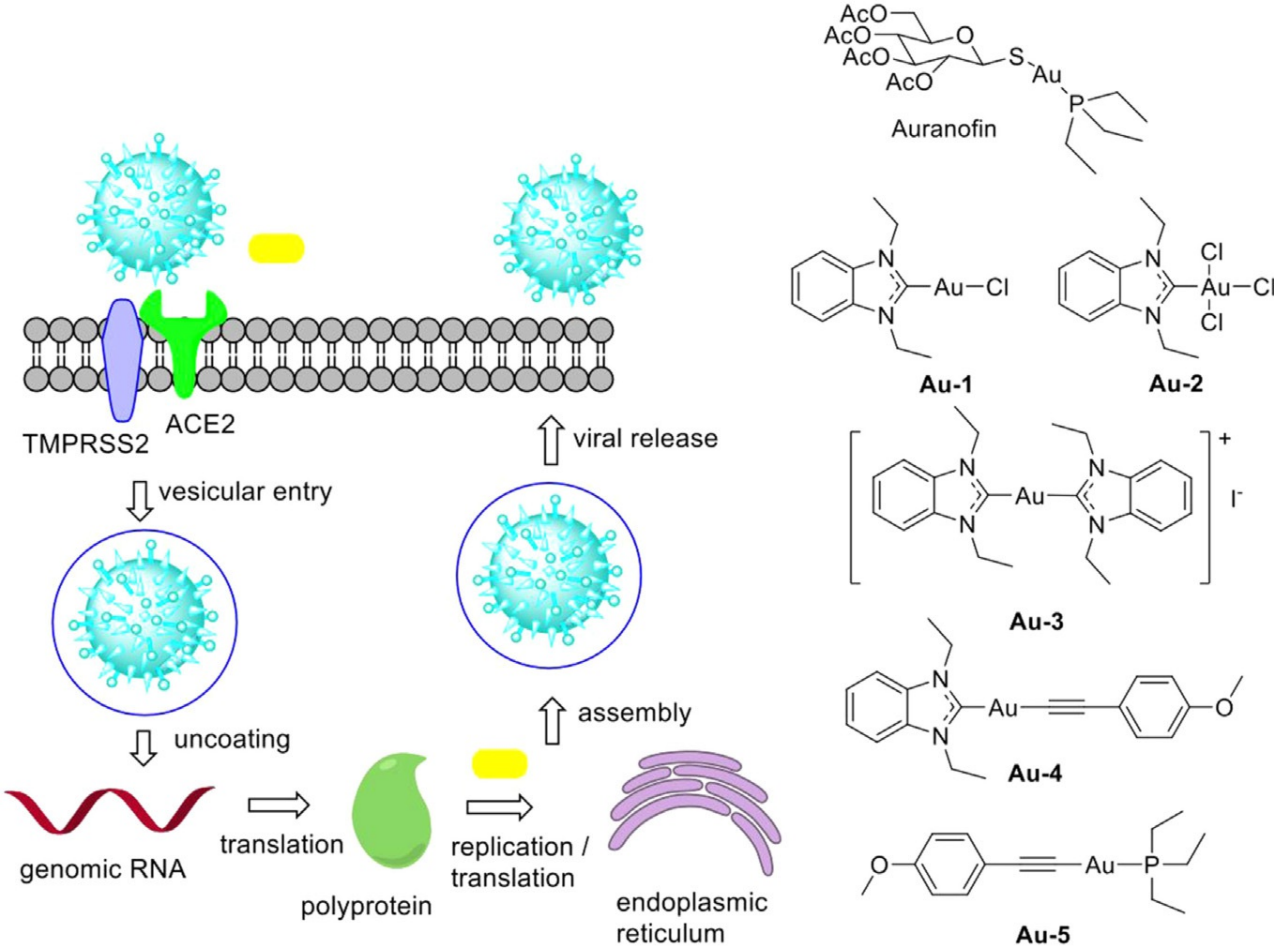
left: simplified SARS‐CoV‐2 life cycle, gold drugs targeting the viral entry and replication are symbolised by golden bars; right: gold metallodrugs used in this study.

The entry of SARS‐CoV‐2 into target cells is facilitated by the spike (S) protein of coronaviruses and mediated by the angiotensin‐converting enzyme 2 (ACE2) as the entry receptor.[^1^, ^4^] The S1 subunit of the SARS‐CoV‐2 spike protein contains the receptor binding domain (RBD). Binding of the RBD to the human ACE2 receptor can be measured by ELISA allowing to evaluate inhibitors of the S protein ACE2 interaction. In this assay, the gold complexes **Au‐1** to **Au‐5** and Auranofin displayed good IC_50_ values in the range of 16–25 μm and were thus slightly more active than the reference drug Chloroquine (IC_50_ value: 31.9 μm).

An essential step in the replication of coronaviruses is the processing of the replicase polyprotein by proteases, such as the papain‐like protease (PLpro), resulting in a number of non‐structural proteins (nsps) that are involved in downstream binding and replication events.[^3^, ^7^, ^8^] SARS‐CoV‐1 PLpro shares 83 % sequence identity with PLpro from SARS‐CoV‐2, structural components of the active sites of the enzymes do not substantially differ. As a cysteine protease PLpro is a likely target for gold‐based drugs, which generally are known to interact with sulfur‐containing molecular targets.

The inhibitory activity of the gold compounds towards PLpro from SARS‐CoV‐1 and SARS‐CoV‐2 was determined by an enzymatic FRET assay. Against PLpro from SARS‐CoV‐1, **Au‐1**, **Au‐2** and **Au‐5** exhibited IC_50_ values in the range of 5–7 μm matching the potency of the reference inhibitor Disulfiram. Complexes **Au‐3** and **Au‐4** were less active with IC_50_ values of 14 μm. Auranofin remained the lowest active gold compound with an IC_50_ value of 25.5 μm (Table 1).

**Table 1:** Inhibition of the spike‐ACE2 interaction and PLpro activity (mean values and standard deviations, *n*=3–4); n.d. not determined. Benzimidazole was used as a negative reference in both assays.

	**spike‐ACE2 (IC_50_, μm)**	**PLpro SARS‐CoV‐1 (IC_50_, μm)**	**PLpro SARS‐CoV‐2 (IC_50_, μm)**
benzimidazole	>100	>100	>100
Chloroquine	31.9±5.4	n.d.	n.d.
Disulfiram	n.d.	6.5±0.4	1.05±0.34
Auranofin	22.2±2.8	25.5±1.2	0.75±0.13
**Au‐1**	19.4±5.7	6.3±1.6	1.04±0.02
**Au‐2**	20.0±2.3	5.5±0.5	1.44±0.22
**Au‐3**	21.3±6.8	14.2±0.3	>100 (53 %)^[a]^
**Au‐4**	25.0±4.2	14.1±2.1	>50 (94 %)^[a]^
**Au‐5**	16.2±2.4	6.7±0.9	0.96±0.07

[a] The percentage indicates the enzyme activity at the highest applied dosage.

Against PLpro from SARS‐CoV‐2, the gold compounds Auranofin, **Au‐1**, **Au‐2** and **Au‐5** as well as the reference inhibitor Disulfiram displayed strong inhibitory activity with IC_50_ values close to 1.0 μm. Notably, **Au‐3** and **Au‐4** were inactive against SARS‐CoV‐2 PLpro with IC_50_ values above 50 μm.

The missing activity of **Au‐3** and **Au‐4** against SARS‐CoV‐2 PLpro and their lower activity against SARS‐CoV‐1 PLpro compared to the other gold compounds indicates that the absence of the more easily exchangeable chlorido, and phosphane ligands prevents a stronger interaction of the gold center with the enzyme. It should also be noted that complexes **Au‐3** and **Au‐4** with their moderate activity against SARS‐CoV‐1 PLpro followed the opposite trend than the other compounds, which were more active against SARS‐CoV‐2 PLpro than against the enzyme from SARS‐CoV‐1.

Cysteine residues in both types of studied SARS‐CoV PLpro are the likely binding sites for gold metallodrugs and this interaction will be facilitated by ligand replacement reactions at the gold center. Importantly, a preprint report confirms that the catalytic cysteine 111 in the active site of SARS‐CoV‐2 PLpro can engage in Michael addition reactions with the β‐carbon of vinyl groups of inhibitors.^[16]^ Coordination of the gold center to this cysteine residue is suggested as a very likely molecular mode of interaction for gold metallodrugs.

Besides the catalytic cysteine PLpro hosts several cysteine residues in a putative labile Zn‐binding domain, which is responsible for correct folding of the protein and stabilization of the local geometry. Ejection of Zn^2+^ from this site represents another likely mechanism for inhibition of PLpro.^[17]^ Of note, the replacement of zinc from zinc‐finger motifs and formation of so called gold‐fingers has been well documented.^[18]^ Interestingly, a very recent paper reports on the dual activity of thiol‐reacting inhibitor Disulfiram as zinc removing agent as well as modifier of the catalytic cysteine of SARS‐CoV‐2 PLpro.^[19]^ As Disulfiram displayed similar activity with the **Au‐1**, **Au‐2** and **Au‐5** against both types of PLpro, it could be speculated that these compounds share such dual activity against the enzyme. Thus we evaluated the ability of the inhibitors to replace zinc ions from PLpro by measuring the released zinc with a zinc selective fluorescent dye.

In the experiments with SARS‐CoV‐1 PLpro, the most efficient inhibitors Disulfiram, **Au‐1**, **Au‐2** and **Au‐5** were effective zinc ejectors, while Auranofin as the lowest active enzyme inhibitor was not an efficient zinc ejector. The zinc removing activity of the moderate SARS‐CoV‐1 PLpro inhibitors **Au‐3** and **Au‐4** was strongly time‐dependent.

In the studies with SARS‐CoV‐2 PLpro all compounds except **Au‐3** and **Au‐4** were efficient zinc ejectors (Figure 2). These results are in excellent agreement with the inactivity of **Au‐3** and **Au‐4** against SARS‐CoV‐2 PLpro as well as the high activity of Auranofin against this enzyme. Taken together, the results of the zinc ejection experiments correlate very well with the activity profile of the gold complexes in the enzymatic FRET assays and explain the differing activities of the complexes against the two types of PLpro.

**Figure 2:**
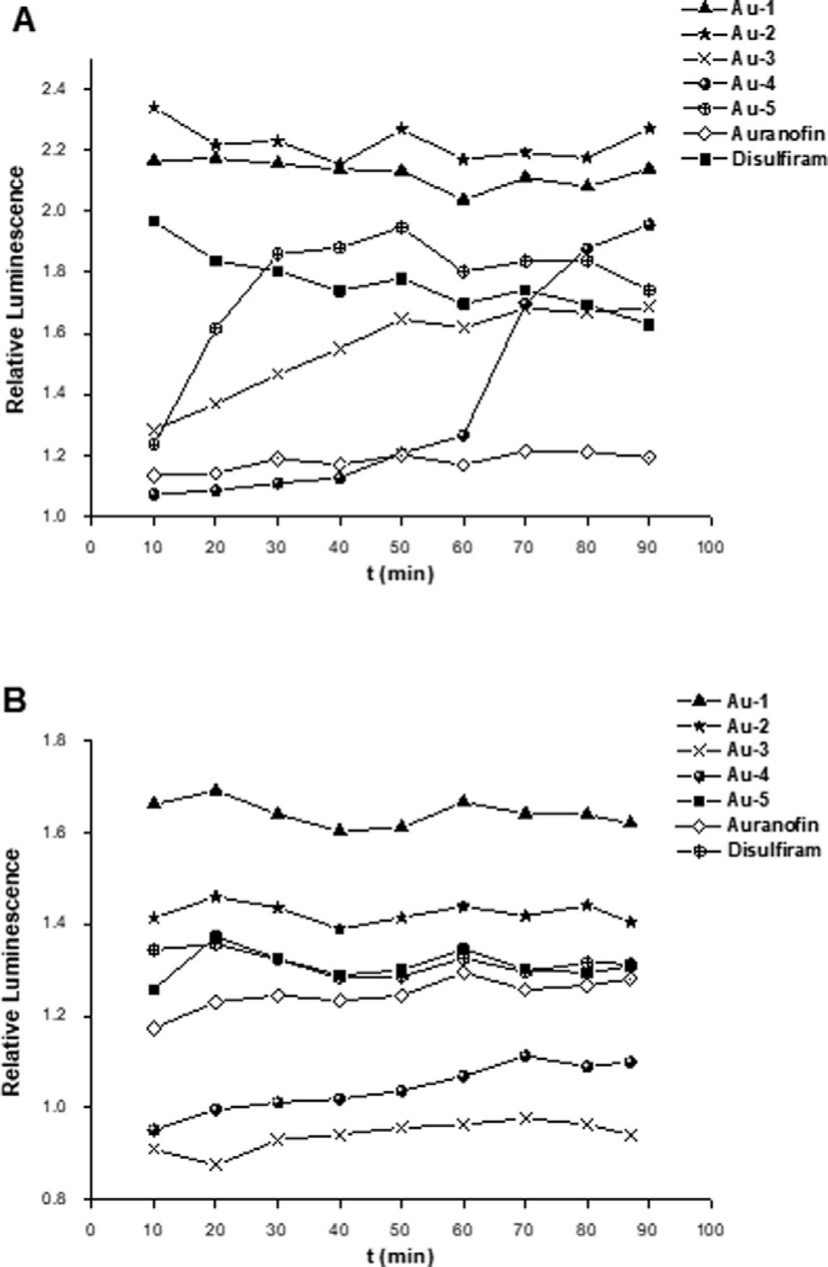
Zinc release from PLpro upon exposure to Disulfiram and gold complexes. The releases Zn^2+^ was detected using the zinc‐specific fluorophore FluoZin^TM^‐3; A) SARS‐CoV‐1 PLpro, B) SARS‐CoV‐2 PLpro.

In conclusion, we have demonstrated that gold complexes can target two relevant pathways in the life cycle of corona viruses. The strongest activity was noted against SARS‐CoV‐2 PLpro with Auranofin and the organometallic gold complexes **Au‐1, Au‐2** and **Au‐5**. The compounds belong thus to the very first potent inhibitors of the target enzyme. Their activity against the enzyme correlates very well with their zinc‐ejecting efficacy.

Notably, the inhibition of the replication of SARS‐CoV‐2 by Auranofin in human cells at low micro molar concentration (below the IC_50_ value for cytotoxicity) was reported very recently.^[20]^ For **Au‐1**, **Au‐3** and **Au‐5** strong cytotoxic activity and effects on the cellular signaling have been reported previously.[^11^, ^12^, ^14^] Such strong effects on host cells at this stage would hamper accurate characterization of possible antiviral effects in cell based models. Hence, a desirable reduction of cytotoxicity against host cells should accompany the ongoing target identification and structure optimization efforts.

The screening of gold and other metal‐based drugs towards relevant SARS‐CoV‐2 molecular targets in combination with a toxicity evaluation is definitely warranted and is ongoing in our laboratories.

## Experimental Section

### General

Chemicals and reagents were obtained from Sigma–Aldrich, TCI, Alfa Aesar and ACROS unless otherwise noted. NMR spectra were recorded on a Bruker DRX‐400 AS or an AV III HD 500 NMR spectrometer; Positive‐ion ESI (electrospray ionization) mass spectra were recorded on a Finnigan MAT95 XL or a LTQ‐OrbitrapVelos linear iontrap coupled with orbitrap mass analyser (ThermoFisher Scientific). Elemental analyses were conducted in a Flash EA1112 apparatus. A VictorTM X4 Perkin–Elmer 2030 multilabel reader was used for the inhibitor assays. Complexes **Au‐1**, **Au‐3** and **Au‐5** were prepared as previously reported.[^11^, ^12^, ^14^]

### (1,3‐Diethyl‐benzimidazol‐2‐ylidene)trichloridogold(III) (Au‐2)

**Au‐2** was prepared according to a reported procedure with modifications.^[21]^ **Au‐1** (98.8 mg, 0.243 mmol, 1.0 equiv) and dichloroiodobenzene (106.7 mg, 0.389 mmol, 1.6 equiv) were dissolved in dichloromethane (4 mL) and the mixture was stirred for 24 h at room temperature under protection from light. Afterwards the solvent was removed under vacuum, the residue was washed three times with *n*‐hexane and diethyl ether each and two times with cold chloroform. The complex **Au‐2** was dried under vacuum at 40 °C. Yield: 80.1 mg (0.168 mmol, 69 %), yellow‐orange powder; ^1^H NMR (500 MHz, [D_6_]DMSO): *δ*=8.05 (dd, ^3^ *J*_H,H_=6.2 Hz, ^4^ *J*_H,H_=3.1 Hz, 2 H, ArH), 7.60 (dd, ^3^ *J*_H,H_=6.2 Hz, ^4^ *J*_H,H_=3.1 Hz, 2 H, ArH), 4.72 (q, ^3^ *J*_H,H_=7.2 Hz, 4 H, CH_2_), 1.52 (t, ^3^ *J*_H,H_=7.2 Hz, 6 H, CH_3_); ^13^C NMR (126 MHz, [D_6_]DMSO): *δ*=147.2 (ArC2_quat_), 132.7 (2 C, ArC3a_quat,_ ArC7a_quat_), 125.7 (2 C, ArC4 + ArC7), 113.0 (2 C, ArC5, ArC6), 43.3 (2 C, CH_2_), 14.4 (2 C, CH_3_); elemental analysis: calcd (%) for C_11_H_14_AuCl_3_N_2_: C 27.67, H 2.95, N 5.87; found: C 27.55, H 2.89, N 5.61; MS(ESI): *m*/*z* 473.0 [*M*−Cl+MeOH]^+^, 209.1 [*M*−AuCl_2_]^+^. Notably, upon oxidation of gold(I) to gold(III) a significant upfield shift of the carbene carbon can be observed in the ^13^C‐NMR spectra.^[21]^ Here the carbene carbon was shifted from 176.3 ppm (**Au‐1**)^[22]^ to 147.2 ppm.

### (1,3‐Diethyl‐benzimidazol‐2‐ylidene)((4‐methoxyphenyl)ethynyl)gold(I) (Au‐4)

1‐Ethynyl‐4‐methoxybenzene (81.3 mg, 0.615 mmol, 1.0 equiv) and potassium hydroxide (207.0 mg, 3.689 mmol, 6.0 equiv) were dissolved in methanol (20 mL) and the mixture was stirred for 10 minutes at 50 °C. Chlorido(1,3‐diethyl‐benzimidazol‐2‐ylidene)gold(I) **Au‐1** (250.0 mg, 0.615 mmol, 1.0 equiv) was dissolved in dichloromethane (1 mL) and added to the mixture. The mixture was stirred for 4 h at 65 °C and for further 60 h at room temperature under protection from light. The solvent was removed under vacuum, the residue was dissolved in dichloromethane and filtered. The solution was washed with a potassium hydroxide solution (20 g/ L), evaporated and dried under vacuum at 40 °C. Yield: 181.2 mg (0.361 mmol, 58 %), yellowish powder; ^1^H NMR (400 MHz, [D_6_]DMSO)=*δ* 7.87–7.78 (m, 2 H, ArH), 7.55–7.43 (m, 2 H, ArH), 7.27–7.19 (m, 2 H, ArH), 6.88–6.80 (m, 2 H, ArH), 4.53 (q, ^3^ *J*_*H,H*_=7.2 Hz, 4 H, CH_2_), 3.74 (s, 3 H, OCH_3_), 1.46 (t, ^3^ *J*_*H,H*_=7.2 Hz, 6 H, CH_3_); elemental analysis: calcd (%) for C_20_H_21_AuN_2_O: C 47.82, H 4.21, N 5.58; found: C 47.91, H 4.19, N 5.43; MS(ESI): *m*/*z* 504.1 [*M*+H]^+^, 875.2 [*M*+NHC‐Au]^+^.

### Spike/ACE2 interaction assay

The inhibition of the spike‐ACE2 interaction was measured using the SARS‐Cov2 Inhibitor Screening Assay kit (Adipogen, Cat. N° AG‐44B‐0007‐KI01). All reagents were used from the same kit during individual experiments and the experiment was performed using the manufacturer's protocol. Briefly, the SARS‐CoV‐2 Spike S receptor binding domain (RBD):Fc (human) (rec.) (SPIKE) was reconstituted to 0.1 mg/mL with deionized water. This was further diluted to a working concentration of 1 μg/mL in phosphate buffered saline (PBS) and used freshly. The assay plate was coated with 100 μL/well of SPIKE, covered with a plastic film and kept at 4 °C overnight. The liquid was aspirated and any remaining liquid was removed by blotting against clean absorbent papers. The plate was blocked using 200 μL of Blocking Buffer per well for 2 h at room temperature. The liquid was aspirated and the wells were washed with 1X Washing Buffer (300 μL *x* 3 times). All liquid was aspirated and excess liquid was removed by blotting against clean absorbent papers. The inhibitors (gold complexes, controls, reference) were diluted in Inhibitor Mix Solution (IMS), which was prepared using ACE2 (human) (rec.) (Biotin) (ACE2) (0.1 mg/mL) to the working concentration of 0.5 μg/mL in 1X ELISA Buffer. The stock solution of the inhibitors was made in DMSO and the final DMSO concentration in the wells was 0.5 %. The IMS‐diluted inhibitors were added to the wells (100 μL/well). The final concentrations of the inhibitors were in the range of 1 to 100 μm. The negative control wells were also treated with 0.5 % DMSO in IMS. The plate was covered with a plastic film and incubated at 37 °C for 1 h after which the aspiration/ wash step was repeated. Next, horseradish peroxidase‐labeled streptavidin (HRP) was reconstituted with 100 μL of 1X ELISA Buffer and further diluted to a working concentration by adding 50 μL in 10 mL of 1X ELISA Buffer (1:200 dilution). It was covered with a plastic film and incubated at RT for 1 h. Following this, the aspiration/ wash step as described earlier was repeated. Substrate development was conducted by the addition of 100 μL of ready‐to‐use 3,3’,5,5’‐tetramethylbenzidine (TMB) to each well for 5 minutes at RT. The reaction was stopped by adding 50 μL of a stop solution. The OD was measured at 450 nm using a PerkinElmer Victor X4 microplate reader. The individual absorbance value of the blank well was subtracted from the other absorbance values and the percentage of the remaining activity was calculated with respect to the untreated control values. Data fitting was done using Origin 2018 using sigmoidal fitting with Hill1 fitting curve. All treatments were done in duplicates and two independent experiments were performed.

### SARS‐CoV‐1 and SARS‐CoV‐2 PLpro inhibition

The inhibition of PLpro was determined according to reported protocols with minor modifications.^[23]^ The inhibitor compounds were prepared as stock solutions in DMSO and diluted hundredfold with HEPES buffer (50 mm HEPES, pH 7.5, 0.1 mg mL^−1^ bovine serum albumin, 0.1 % Triton‐X100) to micromolar concentrations. Volumes of 50 μL of 350 nm His_6_‐SARS‐CoV‐1 PLpro (SouthBayBio) or of 200 nm SARS‐CoV‐2 PLpro (Elabscience) in HEPES buffer or blank HEPES buffer (negative control) were added to the wells of a black 96‐well microtiter plate (Nunclon, Nunc). Volumes of 50 μL of the inhibitor solutions or 1 % DMSO in HEPES buffer (positive control) were added. The resulting solutions (175 nm SARS‐CoV‐1 PLpro or 100 nm SARS‐CoV‐2 PLpro 0.5 % DMSO, 0.1–100 μm test compound or blank HEPES buffer) were mixed and incubated at 37 °C for one hour. A volume of 100 μL of 100 μm Z‐Arg‐Leu‐Arg‐Gly‐Gly‐AMC (Bachem Bioscience) was added to all wells. The resulting solutions were mixed and the fluorescence emission was measured immediately every 30 s for 10 min (λ_exc_=355 nm; λ_em_=460 nm) at 37 °C using a Victor^TM^ X4 Perkin Elmer 2030 multilabel reader. The increase of emission over time followed a linear trend (r^2^ >0.97) and the enzymatic activities were calculated as the slope thereof. The IC_50_ values were calculated as the concentration of the inhibitor that was required to decrease the enzymatic activity to 50 % of the positive control. The wells containing the negative control were used to confirm the absence of false positive results by reaction of the inhibitor compound with the fluorogenic substrate.

### Zn‐ejection assays with SARS‐CoV‐1 and SARS‐CoV‐2 PLpro

To determine if the inhibitors are Zn‐ejecting agents the presence of the Zn^2+^ cation in solution was measured according to a recently published preprint.^[19]^ The inhibitor compounds were prepared as stock solutions in DMSO and diluted hundredfold with HEPES buffer (50 mm HEPES, pH 7.5) to 100 μm concentrations. Volumes of 50 μL of 1 μm His_6_‐SARS‐CoV‐1 PLpro (SouthBayBio) or SARS‐CoV‐2 PLpro (Elabscience) in HEPES buffer or blank HEPES buffer (control for false positive results) were added to the wells of a black 96‐well microtiter plate (Nunclon, Nunc). Volumes of 50 μL of the inhibitor solutions or 1 % DMSO in HEPES buffer (control) were added. The resulting solutions (500 nm PLpro SARS‐CoV‐1 or PLpro SARS‐CoV‐2, 0.5 % DMSO, 50 μm test compound or blank HEPES buffer) were mixed. A volume of 100 μL of 2.0 μm of zinc‐specific fluorophore FluoZin^TM^‐3 (Invitrogen/LifeTechnologies) was added to all wells. The resulting solutions were mixed and the fluorescence emission was measured after 10 min every 10 min for 90 min (λ_exc_=485 nm; λ_em_=535 nm) at 37 °C using a Victor^TM^ X4 Perkin Elmer 2030 multilabel reader. The relative fluorescence was calculated by dividing the absolute fluorescence emission of the well containing the inhibitor by the absolute fluorescence of the respective well containing the enzyme but no inhibitor (control). Wells containing the inhibitor but no enzyme were used to check for false positive results. None of the tested compounds showed false positive results.

## Conflict of interest

The authors declare no conflict of interest.
